# NUMERACY SKILL USE AND SKILL PROFICIENCY AMONG OLDER WORKERS IN THE UNITED STATES

**DOI:** 10.1093/geroni/igae098.3102

**Published:** 2024-12-31

**Authors:** Takashi Yamashita, Donnette Narine, Runcie Chidebe, Phyllis Cummins, Jenna Kramer, Rita Karam

**Affiliations:** University of Maryland Baltimore County, Baltimore, Maryland, United States; University of Maryland Baltimore County, Baltimore, Maryland, United States; Miami University, Oxford, Ohio, United States; Miami University, Oxford, Ohio, United States; RAND Corporation, Arlington, Virginia, United States; RAND Corporation, Santa Monica, California, United States

## Abstract

Recent studies linked numeracy—the ability to understand numeric information -- and a variety of economic well-being outcomes (e.g., employment, income). However, aging is generally associated with lower numeracy skills, and in turn, older workers may face disadvantages in the labor market. The key to maintaining and promoting numeracy skills over the life course is continuous use in everyday life as well as at work. Yet relatively little is known about how older workers use their numeracy skills. Nationally representative data of older workers aged 50 years and older (n = 1,760) were obtained from the 2012/2014/2017 U.S. Program for the International Assessment of Adult Competencies (PIAAC) restricted-use-file pooled data. Latent class analysis was conducted to identify underlying numeracy skill use patterns. Results showed that three classes or subgroups with distinctive numeracy skill use patterns were identified. Subgroup 1 (infrequent users) only used specific and basic numeracy skills, such as calculating prices and costs at home. Subgroup 2 (occupational users) used a variety of numeracy skills, such as preparing graphs and tables, and using formulas, mostly at work. Subgroup 3 (ubiquitous users) used a variety of basic to advanced numeracy skills both at work and at home. Subgroups 1, 2, and 3 had significantly different numeracy skill proficiency scores, 247, 265, and 280 out of 500 points, respectively. Detailed demographic and socioeconomic characteristics were also examined by the identified latent classes to inform education interventions and policies to promote numeracy skills and in turn, economic well-being of older workers.

